# Normocellular CSF in herpes simplex encephalitis

**DOI:** 10.1186/s13104-016-1922-9

**Published:** 2016-02-15

**Authors:** Abhinbhen W. Saraya, Supaporn Wacharapluesadee, Sininat Petcharat, Nuntaporn Sittidetboripat, Siriporn Ghai, Henry Wilde, Thiravat Hemachudha

**Affiliations:** WHO-CC for Research and Training on Viral Zoonoses, Division of Neurology, Department of Medicine, Faculty of Medicine, Neuroscience Centre for Research and Development, Chulalongkorn University and King Chulalongkorn Memorial Hospital, Rama 4 Road, Pathumwan, Bangkok, 10330 Thailand; Division of Infectious Disease, Department of Medicine, Faculty of Medicine, Chulalongkorn University and King Chulalongkorn Memorial Hospital, Bangkok, Thailand

**Keywords:** Encephalitis, Viral encephalitis, Herpes simplex encephalitis, Herpes simplex virus

## Abstract

**Background:**

Herpes simplex virus (HSV) is the most common cause of sporadic encephalitis worldwide. The high mortality rate (70–80 %) of herpes simplex encephalitis (HSE) can be reduced to 20–30 % by antiviral therapy. However, normocellular CSF can lure physicians to look for non-infectious causes, resulting in delayed treatment. This study aimed to investigate, characterize and differentiate HSE patients, with normocellular and pleocytosis CSF, according to neuroimaging patterns, underlying disease, CSF viral load and clinical outcome. Patients with proven (by PCR positive CSF) or presumed viral infections of the CNS admitted to King Chulalongkorn Memorial Hospital between January 2002 and 2011 were analyzed.

**Results:**

HSV was detected in the CSF of 43 patients but only 23 patients had encephalitis. Among these 23 patients, 6 cases (26.1 %) had normal CSF WBC (<5 cells/mm^3^). One patient in this normocellular CSF group had HIV infection. Although this patient had low CD4 counts (<200 cells/mm^3^), the peripheral WBC counts showed only mild leukopenia. The CSF HSV viral load in the pleocytosis group was higher than the normocellular group, with an average of 12,200 vs 3027 copies/ml respectively. There was no correlation between the viral load and the clinical outcome. With respect to neuroimaging, 4 (66.7 %) patients in the normocellular group had unremarkable/non-specific results.

**Conclusions:**

Normocellular CSF in HSE is not rare, and can be seen in normal as well as immunocompromised hosts. Clinicians should not exclude CNS infection, especially HSE, merely based on the absence of CSF pleocytosis and/or unremarkable neuroimaging study.

## Background

Herpes simplex encephalitis (HSE) caused by herpes simplex virus (HSV) is the most common sporadic viral encephalitis among adults worldwide [1, 2]. It has been responsible for up to 20 % of viral encephalitis cases [3]. This disease has a bimodal distribution, where one-third of the patients are under the age of 20, while the remaining are over the age of 50 [4]. Peak incidence is around 60–64 years of age. Neither gender, season, nor immune status of a patient affects its occurrence. The high mortality rate of HSE (70–80 %) can be reduced to 20–30 % by acyclovir therapy. Acyclovir should be taken within 2 days of neurological symptom’s onset as delayed treatment can result in severe morbidity and mortality in approximately one-third of the patients [5, 6].

HSV-1 is responsible for more than 90 % of HSE in adults [7]. Recent studies have demonstrated many cases of HSE that resulted from HSV-2 [8, 9]. HSV can also be associated with myelitis, radiculitis or both. HSV’s preferred areas are the limbic structures, the medial temporal cortex and the orbito-frontal regions, which are all innervated by branches of olfactory and trigeminal nerves. HSV is contracted via contact with the mucosal surface of mouth, eye or genitalia, and has been speculated to subsequently enter the CNS via any nearby sensory nerve ending into these nerves and the corresponding ganglia. Viral entry into the CNS via olfactory tract has been substantiated by numerous animal experiments [10, 11]. However, the neuropathology of a mouse brain is more diffused compared to the well-defined fronto-temporal distribution in a human brain.

HSV can reside in the nerve and can either replicate or establish a latent infection [1]. Its DNA can be detected in the trigeminal ganglion of previously infected patients. At the time of reactivation, latent HSV in the trigeminal ganglia can travel along the tentorial nerve that innervates the meninges of the anterior and the middle cranial fossa. HSV genomes have also been found in olfactory bulb, pons, and the medulla of autopsied brains of normal individuals [12]. Early gross brain pathology of most HSE cases showed hemorrhagic necrosis of the medial or the inferior temporal lobe. These lesions were mainly bilateral and asymmetrical in distribution. Sub-frontal and insular regions were damaged in the latter phase [13]. Brain parenchyma in such regions of HSE patients, who died after 2 weeks of exhibiting HSE, revealed liquefaction necrosis.

The cerebral inflammation from glial cells activation, due to the presence of HSV, includes increase in expression of pattern recognition receptors, especially Toll-like receptors (TLRs) and interferon regulatory factors (IRFs) [4]. Genetic factors, such as deficiency of Toll-like receptor-3 (TLR3), can predispose an individual (especially children and young adults) to HSE [10, 11, 14]. Mutation of TLR3 and TLR3 pathway genes such as UNC93B1, TRIF, TRAF3, and TBK1 can result in the defective production of interferon (IFN). Insufficient amount of IFN-β and IFN-λ leads to an increase in viral replication and enhances cell death. Relapse of HSE tends to occur more frequently in those with abnormal TLR3 immunity. However, these mutations were attributed to only 5 % of HSE in children and young adults [11].

The characteristic symptoms of HSE are abnormal behavior/psychosis (66 %), confusion/disorientation (81 %) and speech disturbance (66 %), which are justifiable due to the preferential involvement of the frontal and temporal lobes. Other symptoms of HSE include fever (76–90 %), headache (70–90 %) and seizure (50–55 %) [5, 15]. However, these symptoms are not unique as they can be present in encephalitis caused by other viruses [16, 17]. Moreover, these symptoms can also be found in immune-mediated encephalitis, in particular the anti-NMDA receptor encephalitis, which can mimic or occur as a relapse encephalitis after the first episode of HSE [18].

Hence, diagnosis of HSE requires an integration of clinical presentations, neuroimaging, and laboratory tests, especially molecular diagnostic techniques. The current gold standard confirmatory test for HSE is polymerase chain reaction (PCR) to detect HSV DNA in the CSF, whose sensitivity is approximately 96 % with a specificity of 99 % in experienced laboratories [19]. For high accuracy of PCR, specimens should be analyzed within 2–10 days after neurological onset [2].

HSE patients are presumed to have evidence of inflammatory response in CNS such as CSF pleocytosis or abnormal neuroimaging. CSF pleocytosis in HSE has been found in more than 90 % of the patients, with an average WBC count of 100–200 cells/mm^3^ [2, 20]. Majority of the cases (80 %) had mild to moderately elevated CSF protein levels (~100 mg/dl), with normal glucose. Detection of red blood cells in the CSF was not an indicator of HSV associated CNS infection [19], but may represent a poor predictor [21]. CSF examination by real-time PCR to detect HSV DNA was validated as a new gold standard for the diagnosis of HSE in 2004 [2, 19, 22]. However, its value in determining viral amount has been controversial [21]. Further, there have been increasing case reports of normal WBC counts (either normocellular or acellular) in the CSF of normal and immunocompromised HSE patients, and some were associated with unremarkable/nonspecific magnetic resonance imaging (MRI) findings [15–17, 23].

## Methods

Patients with proven (by PCR positive CSF) or presumed viral infections of the CNS admitted to King Chulalongkorn Memorial Hospital (KCMH), Bangkok, Thailand, between January 2002 and 2011 were analyzed. Encephalitis was defined as clinical evidence of brain parenchymal dysfunction [6], which could not be explained by metabolic, electrolyte imbalance, bleeding disorders or systemic immune disorders such as systemic lupus erythematosus (SLE), Behcet’s disease or Sjogren syndrome. Bacterial, Rickettsial, fungal, tuberculosis and parasitic causes were excluded by appropriate clinical and laboratory examinations. The diagnosis of HSE was based on the clinical features of encephalitis with the exclusion of other mimics as described above, and the presence of HSV DNA in the CSF.

Retrospective study included those admitted in the years 2002–2009 which did not require ethical approval or written informed consent based on KCMH ethics committee IRB 986/2555. Study on patients admitted in the years 2010–2011 was approved by the KCMH ethics committee (Reference no. 015/2011). Informed consent was obtained from these patients in written form; in cases where the patient was unable to communicate or was underage, consent (and assent where applicable) was obtained from a family member, parent or guardian.

All encephalitis patients with presumed viral causes had their CSF examined by PCR for HSV, Varicella zoster virus (VZV), Epstein-Barr virus (EBV), Cytomegalovirus (CMV) and enteroviruses. Paraneoplastic and autoimmune panels as previously described [24] were tested in one retrospective patient with normocellular CSF and unremarkable brain MRI examination.

### PCR technique for HSV DNA

Qualitative real-time PCR [25] was performed on specimens before 2006, where 5 μl of extracted DNA was used for amplification and detection. Specimens received year 2007 onwards, quantitative assay was performed using *artus*^®^ HSV-1/2 LC PCR kit (Qiagen Inc., Valencia, CA, USA). DNA from 0.1 to 1 mL of CSF was extracted using Nuclisens^®^ extraction kit (bioMérieux, Boxtel, The Netherlands). The amplification was undertaken using a LightCycler^®^ instrument (Roche Diagnostics, Germany). HSV-1 and -2 DNA PCR product was differentiated by melting curve analysis in the LightCycler^®^ PCR instrument. The standard curve was generated from four quantitation standards of HSV (10, 100, 1000 and 10,000 copies/μl) supplied by the manufacturer. Preparation of PCR assay, PCR profile and data analysis was conducted according to the manufacturer’s protocol. The internal control supplied by the manufacturer was included in all reactions to check for possible PCR inhibition. The analytical detection limit of the PCR Kit was 1 copy/μl (p = 0.05) (Qiagen Inc., CA, USA). For qualitative assay, all steps were the same as quantitative assay, except that the standard curve was not used for calculation, and only one standard positive control was performed along with the assayed sample.

## Results

### Patient data

There were 413 patients with viral neurological infections during the 10-year period. All had lymphocytic predominance with normal glucose CSF profile. Those with normal CSF cell count were considered encephalitic only when their brain dysfunctions could not be explained by compromised cardiopulmonary function or other causes. One hundred and fifty-eight patients (38.3 %) had viral etiologies confirmed by PCR. HSV was detected in the CSF of 43 patients (27.2 %) (23 encephalitis and 20 meningitis). Among the 23 HSE patients; there was a slight female predominance (1.3:1), ages ranged between 1 and 85 years [17 (73.9 %) between 16 and 60 (an average of 36)]. Most of HSE patients were previously healthy except 2 (8.7 %) with diabetes, 2 (8.7 %) hematologic malignancies, 1 (4.3 %) inactive SLE, 1 (4.3 %) multiple sclerosis (MS) and 1 (4.3 %) seropositive for human immune deficiency virus (HIV). Most of the patients were from central Thailand. The patient with HIV infection was diagnosed after hospitalization with acute HSE. Prior to hospitalization, the patient had been in good health, never received any highly active antiretroviral therapy (HAART), and did not have any opportunistic infection. The patient’s CD4 count was 200 cells/mm^3^. The patient with MS was in remission and received 50 mg of azathioprine daily. Lastly, the patient with inactive SLE had mild thrombocytopenia, but was not on any immunosuppressive drugs or corticosteroids.

### Clinical findings

Of 23 HSE patients, 16 cases (69.6 %) had fever as a prodrome. The most common presenting symptoms were seizures (7/23, 30.4 %), behavioral changes (6/23, 26 %), alteration of consciousness (5/23, 21.7 %), focal neurological deficits (3/23, 13 %), and worsening of headaches (2/23, 8.7 %). All patients received intravenous administration of acyclovir. Six patients (26.1 %) recovered completely (The Modified Rankin Scale: mRS 0–1), 14 (60.9 %) partially recovered (mRS 2–3), and 3 patients (13 %) died. Demographic data, underlying diseases, and presenting symptoms of each group, classified according to CSF WBC count, are presented in Table 1.

**Table 1:** Demographic data and presenting symptoms of HSV encephalitis in patients with CSF pleocytosis and patients with normocellular CSF

Demographic data	CSF pleocytosis group (WBC > 5cells/mm^3^)	Normocellular CSF group (WBC ≤ 5cells/mm^3^)
Number of patients	17	6
Male:female	9:8	1:5
Median age (years) [range]	35 [1–59]	41 [27–85]
Underlying disease		
Normal	13 (76.5 %)	3 (50 %)
HIV infection	0	1 (16.7 %)
Diabetes mellitus	2 (11.8 %)	0
Hematologic malignancy	2 (11.8 %)	0
Multiple Sclerosis (MS)	0	1 (16.7 %)
Systemic Lupus Erythematosus (SLE)	0	1 (16.7 %)
Prodrome		
Fever	1 (5.9 %)	1 (16.7 %)
Fever with headache	9 (52.9 %)	1 (16.7 %)
Fever with myalgia	1 (5.9 %)	1 (16.7 %)
Upper respiratory tract symptoms	1 (5.9 %)	0
Fever with diarrhea	1 (5.9 %)	0
Fever with non-specific rash	1 (5.9 %)	0
Headache	2 (11.8 %)	0
Non-specific	1 (5.9 %)	3 (50 %)
Presenting symptoms		
Worsening headache with/without neck stiffness	2 (11.8 %)	0
Hemiparesis	1 (5.9 %)	0
Facial weakness	1 (5.9 %)	0
Severe spastic body and limbs	1 (5.9 %)	0
Behavioral change and confusion	4 (23.5 %)	3 (50 %)
Seizures	5 (29.4 %)	2 (33.3 %)
Alteration of consciousness	3 (17.6 %)	1 (16.7 %)
Outcome		
Complete recovery	5 (29.4 %)	1 (16.7 %)
Partial recovery	10 (58.8 %)	4 (66.7 %)
Dead	2 (11.8 %)	1 (16.7 %)

### Laboratory findings

All 23 cases had serum creatinine in the range of 0–3 mg/dl and normal liver function test. Complete blood count data was available in 20 patients, of whom 14 (70 %) were within the normal limit, 1 (5 %) had pancytopenia (patient no. 17 in pleocytosis group), and 1 (5 %) had leucopenia (patient no. 6 in normocellular group). Seventeen of 23 patients (73.9 %) had CSF examination within 2–10 days after neurological symptoms onset, the golden period for CSF PCR examination for HSV [7]. CSF findings are detailed in Table 2. CSF protein levels ranged between 14 and 528 mg/dl (mean 108.3 mg/dL), while CSF glucose levels were between 30 and 139 mg/dl (mean 66.2 mg/dl). Seventeen patients (73.9 %) had CSF WBC counts between 6 and 500 cells/mm^3^, with lymphocytic predominance.

**Table 2:** CSF profiles and imaging findings

CSF Profile	CSF pleocytosis group (WBC > 5cells/mm^3^)	Normocellular CSF group (WBC ≤ 5cells/mm^3^)
Days of CSF examination after neurological onset	Day 0—1: 5 (29.4 %) Day 2—10: 12 (70.6 %)	Day 0—1: 1 (16.7 %) Day 2—10: 5 (83.3 %)
CSF protein	0–60 mg/dl: 1 (6.3 %) 61–100 mg/dl: 7 (43.8 %) >100 mg/dl: 8 (50 %) Not available: 1	0–60 mg/dl: 2 (33.3 %) 61–100 mg/dl: 4 (66.7 %) >100 mg/dl: 0
CSF glucose	<30 mg/dl: 1 (6.3 %) 31–60 mg/dl: 7 (43.8 %) >60 mg/dl: 8 (50 %) Not available: 1	<30 mg/dl: 0 31–60 mg/dl 1 (16.7 %) >60 mg/dl: 5 (83.3 %)
Average CSF viral load	12,200 copies/ml (39–11,755,813 copies/ml) (11 cases available: 6 had qualitative test)	3027 copies/ml (59–5970 copies/ml) (4 cases available: 3 had qualitative test)
Brain imaging findings	16 available records	6 available records
Predominant temporal lobe lesions	5 (29.4 %)	1 (16.7 %)
Fronto-temporal lesions	4 (23.5 %)	1 (16.7 %)
Other cortical and subcortical lesions (non fronto-temporal)^a^	3 (18.8 %)	0
Fronto-temporal and thalamic lesions^a^	3 (18.8 %)	0
Normal or Miscellaneous^b^	0	2 (33.3 %)
Normal CT (MRI not performed)	1 (5.9 %)	2 (33.3 %)

^a^Two of the patients (a non fronto-temporal lesion and one with fronto-temporal and thalamic lesions) also had vasculitis, visualized using magnetic resonance angiography

^b^Miscellaneous refers to non-specific lesions such as non-specific white matter changes, aging brain etc

Six cases (26.1 %), with CSF examinations within 10 days after onset of illness (an average of 3 days), had normal CSF WBC (<5 cells/mm^3^). One patient in this normocellular CSF group (16.7 %) had HIV infection. Although this patient had low CD4 counts (<200 cells/mm^3^), the peripheral WBC counts were 3150 and 3570 cells/mm^3^ in two consecutive tests.

Seventeen patients had CSF pleocytosis with CSF WBC ranging between 15 and 730 cells (mean 179 cells/mm^3^), 3 of whom had CSF WBC count greater than 500 cells/mm^3^. Thirteen patients (76.5 %) were previously healthy. None of them had HIV infection. CSF HSV viral load in this group was higher than the normocellular group, with an average of 12,200 vs 3027 copies/ml respectively. Of all 14 specimens received after 2007, 10 cases (2 normocellular, 8 pleocytosis) were positive for HSV1 and 4 cases (1 normocellular, 3 pleocytosis) were positive for HSV2 encephalitis. Other CSF parameters are described in Table 2.

### Imaging findings

Twenty-two patients had complete neuroimaging (either computer tomography (CT) or MRI) records. Eleven (50 %) patients had the predominant temporal or fronto-temporal lobe lesions and 2 (9.1 %) had fronto-temporal lesion coexisting with thalamic lesions. Another two patients had cortical/subcortical (non fronto-temporal) lesions. Two patients (9.1 %) had bead-like appearance resembling vasculitis on MR angiography, whereas 5 (22.7 %) had normal or non-specific findings. Brain imaging data altogether with CSF HSV viral load of normocellular and pleocytosis groups are presented in Tables 3 and 4.

**Table 3:** Individualized data of the CSF pleocytosis group of HSE patients

Patient no.	Underlying disease	CSF WBC (cells/mm^3^)	Imaging pattern (MRI)	CSF HSV viral load (copies/ml)	Outcome
1	Normal	230	Fronto-temporal lesions	16,975	Complete recovery
2	Normal	350	Fronto-temporal lesions	2214	Complete recovery
3	Normal	70	Fronto-temporal lesions	Qualitative PCR	Complete recovery
4	Normal	730	Temporal lobe lesions	11,755,813	Partial recovery
5	Normal	22	Fronto-temporal and thalamic lesions	500,000	Partial recovery
6	Normal	90	Temporal lobe lesions	71,875	Complete recovery
7	Hematologic malignancy	147	Temporal lobe lesions	30,750	Complete recovery
8	Normal	75	Fronto-temporal and thalamic lesions	12,200	Partial recovery
9	DM	510	Other cortical and subcortical lesions	3325	Dead
10	Normal	40	Normal CT	1491	Partial recovery
11	Hematologic Malignancy	40	Other cortical and subcortical lesions	740	Dead
12	Normal	60	Fronto-temporal and thalamic lesions	39	Partial recovery
13	DM	15	N/A	Qualitative PCR	Partial recovery
14	Normal	600	Temporal lobe lesions	Qualitative PCR	Partial recovery
15	Old ischemic stroke	22	Fronto-temporal lesions	Qualitative PCR	Partial recovery
16	Normal	30	Temporal lobe lesions	Qualitative PCR	Partial recovery
17	Normal	20	Other cortical and subcortical lesions	Qualitative PCR	Partial recovery

**Table 4:** Individualized data of the normocellular CSF group of HSE patients

Patient no.	Underlying disease	CBC	Imaging pattern (MRI)	CSF HSV viral load (copies/ml)	Outcome
1	Systemic Lupus Erythematosus	Thrombocytopenia	Normal MRI	5970	Dead
2	Multiple Sclerosis	Normal	Old white matter lesions	3144	Partial recovery
3	Normal	Leukocytosis	Fronto-temporal lesion	2910	Complete recovery
4	Normal	Normal	Temporal lobe lesion	59	Partial recovery
5	Normal	Normal	Normal CT	Qualitative PCR	Partial recovery
6	HIV infection	Leukopenia	Normal CT	Qualitative PCR	Partial recovery

## Discussion

In this study, 6 of 23 HSE patients (26.1 %) had normal CSF WBC counts as shown in Tables 1 and 2. All normocellular patients had CSF protein levels less than 100 mg/dl, whereas half of the patients in the pleocytosis group had CSF protein levels greater than 100 mg/dl. All brain MRIs of the 15 patients in the pleocytosis group, and 2 of 4 patients in the normocellular group showed abnormal lesions. Two normocellular patients had normal brain MRI and high viral load, 5970 and 3144 copies/ml respectively. One of them had inactive SLE with drowsiness, while the other had MS (in remission) with behavioral change (Table 4). Two patients in the normocellular group had CT scan, which might not have been sensitive enough to detect abnormalities, as compared to MRI. However, both did not have any other explainable causes. There was no data on CSF HSV viral load in both cases as only qualitative PCR data was available. One patient was first diagnosed as HIV positive at the time of admission with HSE, with no history of previous opportunistic infections. HIV viral load was not performed in the CSF and data on viral load in the blood was not available. Although HIV encephalitis could not be excluded, such acute presentation suggests otherwise. Retrospective autoantibody test was negative. Both patients had partial recovery with acyclovir treatment.

Our study showed that HSE with normocellular CSF is more common than what was generally known in the past. The CSF protein levels, number of cases with abnormal neuroimaging, and CSF viral loads in the pleocytosis group were higher when compared to the normocellular group. It was noted that CSF viral load was up to four times higher in CSF pleocytosis group than normocellular group (an average of 12,200 vs 3027 copies/ml), potentially due to increased inflammatory response in pleocytosis group. Viral load alone may not entirely explain the magnitude of CSF cellular response, as patients with nonspecific MRI in the normocellular group also had high copies of viral load. When looking at individual data in normocellular group (Table 4), the patient with the highest viral load (5970 copies/ml) had normal MRI and CSF protein level of 14 mg/dl. Why some HSE patients have normocellular CSF, or normal/nonspecific MRI remains a mystery. Whether viral load and/or genetic factors influence inflammatory responses in the CSF or abnormal MRI findings is not yet known. Moreover, viral load may not affect the outcome, as patients in normocellular group with lower viral load were, in fact, more inclined to be more severely affected by the disease (one died, four partially recovered, one complete recovery) compared to the pleocytosis group, which had higher viral loads on average (two died, ten partially recovered, five complete recovery). This conformed to a previous study which reported no correlation between CSF viral load and clinical outcome in HSE patients [21]. Further study in the CSF of HSE patients which compares the inflammatory responses in both pleocytosis and normocellular group is needed to elucidate the pathophysiology of HSE.

A retrospective study of 35 HSE patients in Spain over a 15 year period revealed that 8 (22.8 %) had normal CSF WBC counts [26]. However, all patients in this Spanish study had neuroimaging abnormalities but only 92 % of patients had PCR positive for HSV. Furthermore, a large encephalitis study in Turkey, which recruited 106 patients between 2001 and 2012, revealed approximately 15 % of HSE patients had acellular and normocellular CSF [5]. The Turkish study also revealed that as many as 5 % had no MRI lesions.

Our study had some limitations, such as small sample size, and lack of complete data in some subjects. However, the study also revealed important data that directly affects the clinical management of HSE. An absence of CSF pleocytosis and/or MRI abnormalities does not exclude infectious encephalitis, in particular HSE. Additionally, examination of HSV DNA by PCR in the CSF is mandatory in the management of encephalitis. Our study indicates that relying on the CSF pleocytosis and/or presence of classical MRI features of HSE may result in a delay in diagnosis and treatment. A larger study is further required to confirm results statistically and analyze inflammatory responses in normocellular HSE, when compared to pleocytosis group, to better understand the pathophysiology of HSE.

## Conclusion

Absence of CSF pleocytosis in HSE has been described as a rare finding in the past, which can distract physicians from the veritable diagnosis. Our study intended to demonstrate the extent to which normal CSF WBC findings are found in encephalitis associated with HSV. It is not uncommon, and can be found in previously healthy patients. Therefore, clinicians should not exclude CNS infection (especially HSE) based on the absence of CSF pleocytosis and normal neuroimaging.

## Abbreviations

CD4: cluster of differentiation 4
CMV: Cytomegalovirus
CNS: central nervous system
CSF: cerebrospinal fluid
CT: computed tomography
DNA: deoxyribonucleic acid
EBV: Epstein–Barr virus
HIV: Human immunodeficiency virus
HSE: Herpes simplex encephalitis
HSV: Herpes simplex virus
IFN: interferon
IRB: Institutional Review Board
IRF: interferon regulatory factor
KCMH: King Chulalongkorn Memorial Hospital
MR: magnetic resonance
MRI: magnetic resonance imaging
MS: multiple sclerosis
NMDA: N-methyl-D-aspartate
PCR: polymerase chain reaction
SLE: systemic lupus erythematosus
TANK: TRAF family member-associated NFKB activator
TBK1: TANK-binding kinase 1
TIR: toll-interleukin 1 receptor
TLR: toll-like receptors
TNF: tumour necrosis factor
TRAF3: TNF receptor associated factors
TRIF: TIR-domain-containing adapter-inducing interferon-β
UNC93B1: Unc-93 homolog B1
VZV: Varicella zoster virus
WBC: white blood cell

## References

[1] Kennedy PG, Steiner I (2013). Recent issues in herpes simplex encephalitis. J Neurovirol.

[2] Lim HK (2014). TLR3 deficiency in herpes simplex encephalitis: high allelic heterogeneity and recurrence risk. Neurology.

[3] Granerod J (2010). Causes of encephalitis and differences in their clinical presentations in England: a multicentre, population-based prospective study. Lancet Infect Dis.

[4] Koskiniemi M (1996). Herpes encephalitis is a disease of middle aged and elderly people: polymerase chain reaction for detection of herpes simplex virus in the CSF of 516 patients with encephalitis. The Study Group. J Neurol Neurosurg Psychiatry.

[5] Whitley RJ (1986). Vidarabine versus acyclovir therapy in herpes simplex encephalitis. N Engl J Med.

[6] Solomon T (2012). Management of suspected viral encephalitis in adults–Association of British Neurologists and British Infection Association National Guidelines. J Infect.

[7] Aurelius E (1993). Encephalitis in immunocompetent patients due to herpes simplex virus type 1 or 2 as determined by type-specific polymerase chain reaction and antibody assays of cerebrospinal fluid. J Med Virol.

[8] Moon SM (2014). Comparison of clinical manifestations, outcomes and cerebrospinal fluid findings between herpes simplex type 1 and type 2 central nervous system infections in adults. J Med Virol.

[9] Mateen FJ, Miller SA, Aksamit AJ (2014). Herpes simplex virus 2 encephalitis in adults. Mayo Clin Proc.

[10] Barnett EM (1994). Herpes simplex encephalitis in the temporal cortex and limbic system after trigeminal nerve inoculation. J Infect Dis.

[11] Whitley RJ (1977). Adenine arabinoside therapy of biopsy-proved herpes simplex encephalitis. National Institute of Allergy and Infectious Diseases collaborative antiviral study. N Engl J Med.

[12] Tyler KL (2004). Herpes simplex virus infections of the central nervous system: encephalitis and meningitis, including Mollaret’s. Herpes.

[13] Baringer JR (2008). Herpes simplex infections of the nervous system. Neurol Clin.

[14] Baringer JR, Pisani P (1994). Herpes simplex virus genomes in human nervous system tissue analyzed by polymerase chain reaction. Ann Neurol.

[15] Domingues RB (1997). Evaluation of the range of clinical presentations of herpes simplex encephalitis by using polymerase chain reaction assay of cerebrospinal fluid samples. Clin Infect Dis.

[16] Kennedy PG, Chaudhuri A (2002). Herpes simplex encephalitis. J Neurol Neurosurg Psychiatry.

[17] Whitley RJ, Gnann JW (2002). Viral encephalitis: familiar infections and emerging pathogens. Lancet.

[18] Steiner I, Tyler KL (2014). The toll (like receptor 3) to the pathogenesis of herpes simplex encephalitis. Neurology.

[19] Steiner I (2012). EFNS-ENS guidelines for the use of PCR technology for the diagnosis of infections of the nervous system. Eur J Neurol.

[20] Lafaille FG (2012). Impaired intrinsic immunity to HSV-1 in human iPSC-derived TLR3-deficient CNS cells. Nature.

[21] Desena A (2014). Herpes simplex encephalitis as a potential cause of anti-N-methyl-D-aspartate receptor antibody encephalitis: report of 2 cases. JAMA Neurol.

[22] Gkrania-Klotsas E, Lever AM (2008). Herpes simplex I encephalitis presenting as a brain haemorrhage with normal cerebrospinal fluid analysis: a case report. J Med Case Rep.

[23] Mook-Kanamori B, van de Beek D, Wijdicks EF (2009). Herpes simplex encephalitis with normal initial cerebrospinal fluid examination. J Am Geriatr Soc.

[24] Schoonman GG (2012). Herpes simplex virus encephalitis without cerebrospinal fluid pleocytosis is not unusual. J Am Geriatr Soc.

[25] Kessler HH (2000). Detection of Herpes simplex virus DNA by real-time PCR. J Clin Microbiol.

[26] Riera-Mestre A (2009). Adult herpes simplex encephalitis: fifteen years’ experience. Enferm Infecc Microbiol Clin.

